# Lower Limb Resistance Training in Individuals With Parkinson's Disease: An Updated Systematic Review and Meta-Analysis of Randomized Controlled Trials

**DOI:** 10.3389/fneur.2020.591605

**Published:** 2020-11-13

**Authors:** Xiaoyan Li, Jie He, Jie Yun, Hua Qin

**Affiliations:** ^1^Department of Endocrinology, Clinical Medical College and The First Affiliated Hospital of Chengdu Medical College, Chengdu, China; ^2^Department of Respiratory and Critical Care Medicine, Clinical Medical College and The First Affiliated Hospital of Chengdu Medical College, Chengdu, China; ^3^Nursing Department of Affiliated Hospital of Chengdu University of Traditional Chinese Medicine, Chengdu, China

**Keywords:** resistance training, Parkinson's disease, randomized controlled trial, systematic review, meta-analysis, lower limb gait, physical function

## Abstract

**Objective:** Initial randomized controlled trials (RCTs) and recently released systematic reviews have identified resistance training (RT) as a modality to manage motor symptoms and improve physical functioning in individuals with Parkinson's disease (PD), although the effects are inconsistent. Therefore, we conducted an updated meta-analysis to reassess the evidence of the relationship.

**Methods:** We performed a systematic search of studies reporting the effects of RT in PD available through major electronic databases (PubMed, Medline, Embase, Ovid, Cochrane Library, CNKI, Wanfang) through 20 July 2020. Eligible RCTs were screened based on established inclusion criteria. We extracted data on the indicators of leg strength, balance, gait capacity, and quality of life (QoL) of lower limbs. Random and fixed effects models were used for the analysis of standard mean differences (SMD) or mean differences (MD) with their 95% confidence intervals (CI).

**Results:** Thirty-one papers from 25 independent trials compromising 1,239 subjects were selected for eligibility in this systematic review and meta-analysis. Summarized data indicated that the leg strength increased statistically significant in PD patients (SMD = 0.79, 95% CI 0.3, 1.27, *P* = 0.001), the balance capability was improved statistically significant in PD patients (SMD = 0.34, 95% CI 0.01, 0.66, *P* = 0.04), and QoL statistically significantly improved (MD = −7.22, 95% CI −12.05, −2.39, *P* = 0.003). For gait performance, four indicators were measured, the results as follows: fast gait velocity (MD = 0.14, 95% CI 0.06, 0.23, *P* = 0.001), Timed-up-and-go-test (TUG, MD = −1.17, 95% CI −2.27, −0.08, *P* = 0.04) and Freezing of Gait Questionnaire (FOG-Q, MD = −1.74, 95% CI −3.18, −0.3, *P* = 0.02) were improved statistically significant across trials, while there were no statistically significant improvement in stride length (MD = −0.05, 95% CI −0.12, 0.02, *P* = 0.15) in PD patients.

**Conclusions:** Lower limb RT has positive effects during rehabilitation in individuals with PD in leg strength, QoL, and improve gait performance to a certain extent. RT also could improve balance capacity of patients, although a wide variety of tools were used, and further study is needed to confirm these findings.

## Introduction

Parkinson's disease (PD) is a highly prevalent and progressive degenerative disorder of the nervous system, primarily affecting the middle-aged and elderly. Approximately 1% of people 60 years and older are affected (1–3). The estimated prevalence of PD is expected to double by 2040, with prevalence in USA alone over 1.6 million, due mainly to changing demographic profiles and increasing of life expectancy (4, 5). Clinical symptoms of PD include motor symptoms and non-motor symptoms. The classic tetrad of Parkinson's symptoms includes—resting tremor, bradykinesia, rigidity, and loss of postural reflexes. These motor difficulties bring about decreased muscle strength and increased tension in the lower limbs, further impairing balance performance and seriously affecting standing and walking postural instability (6). Postural instability increases the risk of falling and fear of falling, and often results in increased sedentarism, resulting in poor quality of life (QoL) (7, 8). Non-motor symptoms include anxiety, depression, neuropsychiatric symptoms, and sleep disturbances, also negatively impacting the quality in patients with PD life in addition to the motor symptoms (9, 10).

Presently, treatment for PD is limited to symptomatic management and etiological management remains under development. Exercise training has strong evidence supporting its use to alleviate motor dysfunction and physical discomfort in PD and could improve cognitive function, drug efficacy, sleep patterns, and mitigate depression (11), and has therefore attracted substantial clinical interest. Exercise therapies (12, 13) include passive exercise methods such as acupuncture and massage, and active exercise methods such as Yoga and Tai Chi. The above modalities rely on the guidance of doctors and coaches to vary degrees, and have high requirements for patient compliance. However, some studies have reported resistance training (RT) as a method to increase muscle length and enhance muscle strength by overcoming external resistance, which can be conducted at home after patient training. Compared with other physical training methods, it is more practical and flexible for a wide variety of patient lifestyles (14, 15). Accumulating evidences indicate that RT improves balance, gait performance, and lower limb muscle strength in PD (8–12, 14). A recent study of multicentered, RCT indicated the effectiveness of an 8-week stretching and RT exercises program in 138 PD patients and concluded a clinically significant improvement in motor dysfunction and mobility, with a positive effect of physical and functional capacities (16). Vieira De Moraes Filho et al. (17) also revealed that RT reduces bradykinesia, motor symptoms, and improves functional performance in patients with PD. Currently, the mechanism of RT on functional fitness and activities in persons with PD is unclear, some related studies show that RT improves the antioxidant capacity of patients, induces muscle hypertrophy, increases muscle cross-sectional area, muscle strength, endurance, effectively alters muscle shape, modifies the rates of motor neurons and the synchronization of motor units, as well as improves cognitive function (10, 18–21).

RT has been proven to improve the strength, some physical functionality, and motor function in patients with PD, and is widely used in clinical non-drug treatment for PD (22, 23). However, the results of recent meta-analyses were different, even conflicting, especially in the improvement of gait and balance (24, 25). A previous meta-analysis by Tillman et al. (24) reported that RT increased leg strength but not gait performance or balance in subjects with PD, although the studies included in this review were limited, with limited numbers of patients in each trial (24). Recently, various studies have found improvements in functional capabilities as a result of RT (23, 26). Thus, considering the inconsistent results, our objective was to reassess the existing uncertainty in regard to evidence supporting the effects of RT on gait and balance in subjects with PD by conducting a new systematic review and meta-analysis of extant RCTs.

## Methods

### Search Strategy

**Figure 1:**
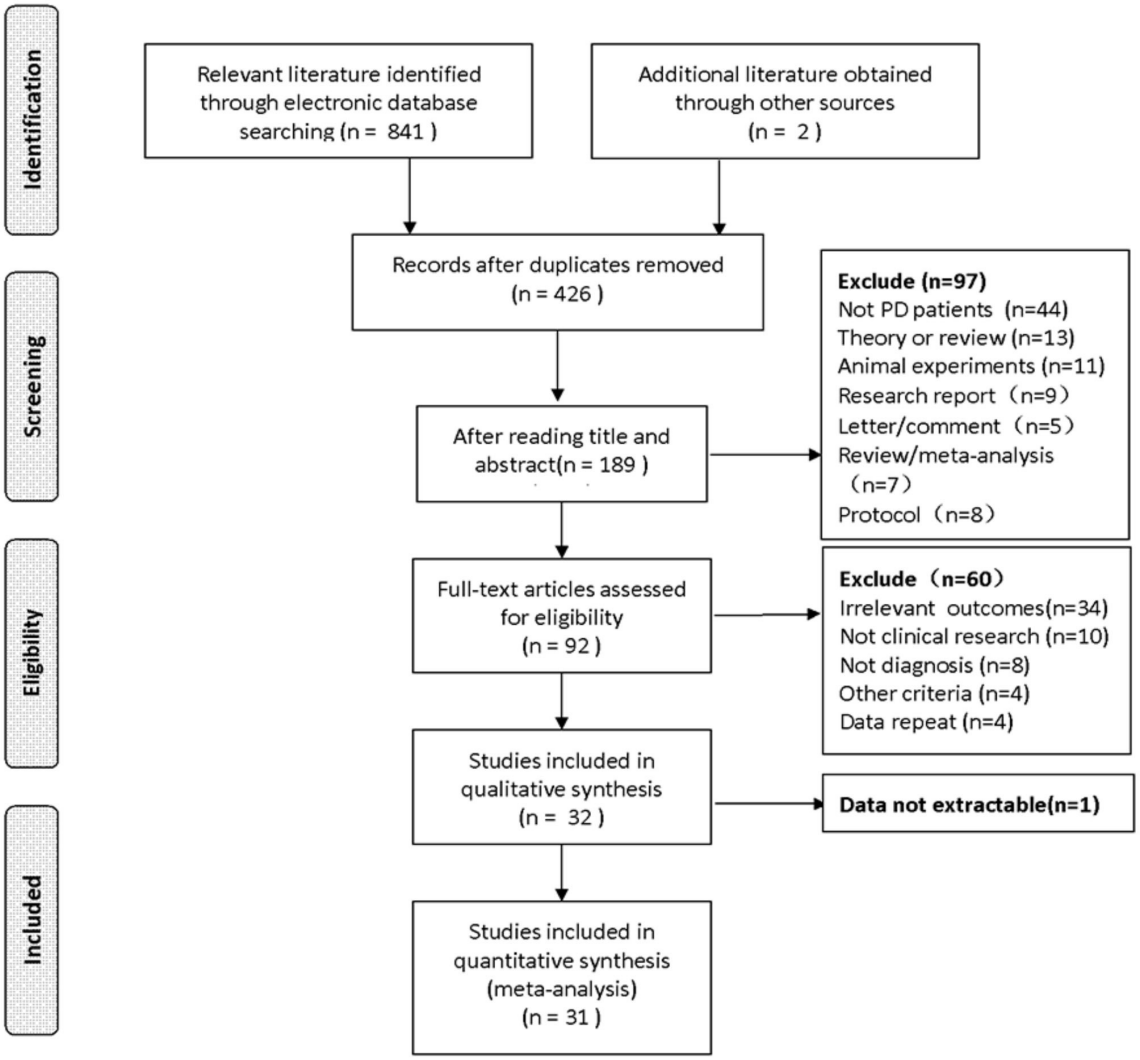
Flow diagram of literature selecting process and results according to the preferred reporting items for the meta-analysis. PD, Parkinson's disease.

We systematically searched available literature reporting on subjects with PD undergoing RT in several electronic databases including PubMed, Medline, Embase, Ovid, Cochrane Library, China National Knowledge Infrastructure (CNKI) and Wanfang until revised: 20 July 2020. Only English and Chinese studies were considered. Search terms among these studies were “Parkinson,” “Parkinson's disease,” “PD,” “Parkinsonism,” “resistance training,” “strength training,” “power training,” and “controlled clinical trials.” References of published articles were also checked during the selection of literature initially retrieved in our study. A flow diagram in Figure 1 displays the retrieval process and results.

### Study Selection and Eligibility Criteria

Studies included in this review were required to meet the following criteria for inclusion: (1) the study utilized an RCT design and assessed the effects of RT in subjects with PD, which included RT against other exercise or no motor intervention. Studies that combined RT with other exercise but could independently assess the effectiveness of RT were also included in the study; (2) RT was focused on the lower limbs; (3) the outcomes considered at least one aspect of strength and physical function, including gait performance, balance, leg strength, or QoL; (4) full text of the article was published in English or Chinese. Studies that met the following conditions were excluded: (1) studies that were conducted on the effects of mixed exercise beyond RT only, and could not solely evaluate the effect of RT in PD; (2) studies for which the full text or main results were unavailable; (3) studies which were case reports, protocols, animal studies, qualitative analyses, reviews, or commentary. Based on the eligibility criteria, XYL and JH independently screened titles and abstracts and read the full text of included literature. If two or more trials were reporting on identical cohorts, the article published most recently, or which was the most comprehensive was retained.

### Data Extraction and Quality Evaluation

The basic data and clinical characteristics were gathered as follows: author, country, publication year, sex ratio, age, sample size, disease stage (Hoehn-Yahr Stage), disease duration, frequency and intervention time, follow-up period, outcome measures, and intervention of different group. Two authors XYL and JH, independently extracted the data and cross-checked the information; discrepancies were resolved through discussion or consultation with another experienced author JY until a consensus was reached. Quality of the included articles was evaluated using the bias risk assessment from the Cochrane collaboration Network, which included whether the random allocation method was correct, whether the allocation was hidden, whether blinding methods were used, whether there was loss of follow-up, the presence of selective reporting biases, or other biases.

### Outcome Measures

The outcome measures adopted in our meta-analysis were direct quantitative scores provided by preliminary studies. We evaluated the efficacy of RT on subjects with PD in the following aspects. First, leg strength in kilograms or Newtons was assessed by one repetition maximum (1-RM) leg press strength, strain gauge or force platform assessing the lower limbs. Second, balance capability was assessed with different tools, including the Berg scale, Fullerton Advanced Balance (FAB) scale, Balance Evaluation Systems Test (BESTest), Mini-best and Coordinated stability test. Third, gait performance was evaluated by stride length, fast gait velocity (which could be measured by 6- or 10-m walking tests, second chronograph and computer), Timed-up-and-go-test (TUG) and freezing of Gait Questionnaire (FOG-Q). Finally, QoL was assessed by the Parkinson's Disease Questionnaire (PDQ-39).

### Statistical Analysis

Revman 5.3 and Stata 12.0 software were utilized to conduct the meta-analysis. Pooled mean difference (MD)/standard mean difference (SMD) and 95% confidence intervals (95% CI) were calculated for continuous variables to compare the different effects of resistance training in subjects with PD between experimental and control groups. The heterogeneity of the results was analyzed by the Cochrane-*Q*-test and *I*^2^-statistic (values of 25, 50, 75%, respectively, represented degrees of heterogeneity at low, moderate, and high levels.). If *P* > 0.1, *I*^2^ < 50% indicated that the studies were homogeneous and a fixed effects model could be adopted; otherwise, a random effects model was necessary. Obvious heterogeneity was processed by sensitivity analysis or only descriptive analysis. The publication bias was analyzed by Egger's test and Begg's test to assess quantitative evaluation results.

## Results

### Search Results

The detailed literature retrieval process in the study can be found in Figure 1. The preliminary search yielded 843 records, which was reduced to 426 articles after the exclusion of duplicates. Two authors screened titles and abstracts, and 237 articles were excluded for irrelevance to the topic. The 189 remaining literature were carefully evaluated by full-text review; 92 articles were considered suitable for the inclusion. After removing articles with irrelevant outcomes or criteria, and 1 article for which reliable data could not be extracted, 31 eligible articles from 25 independent trials were assessed for final eligibility and included in this meta-analysis.

### Characteristics of Included Studies

**Figure 2:**
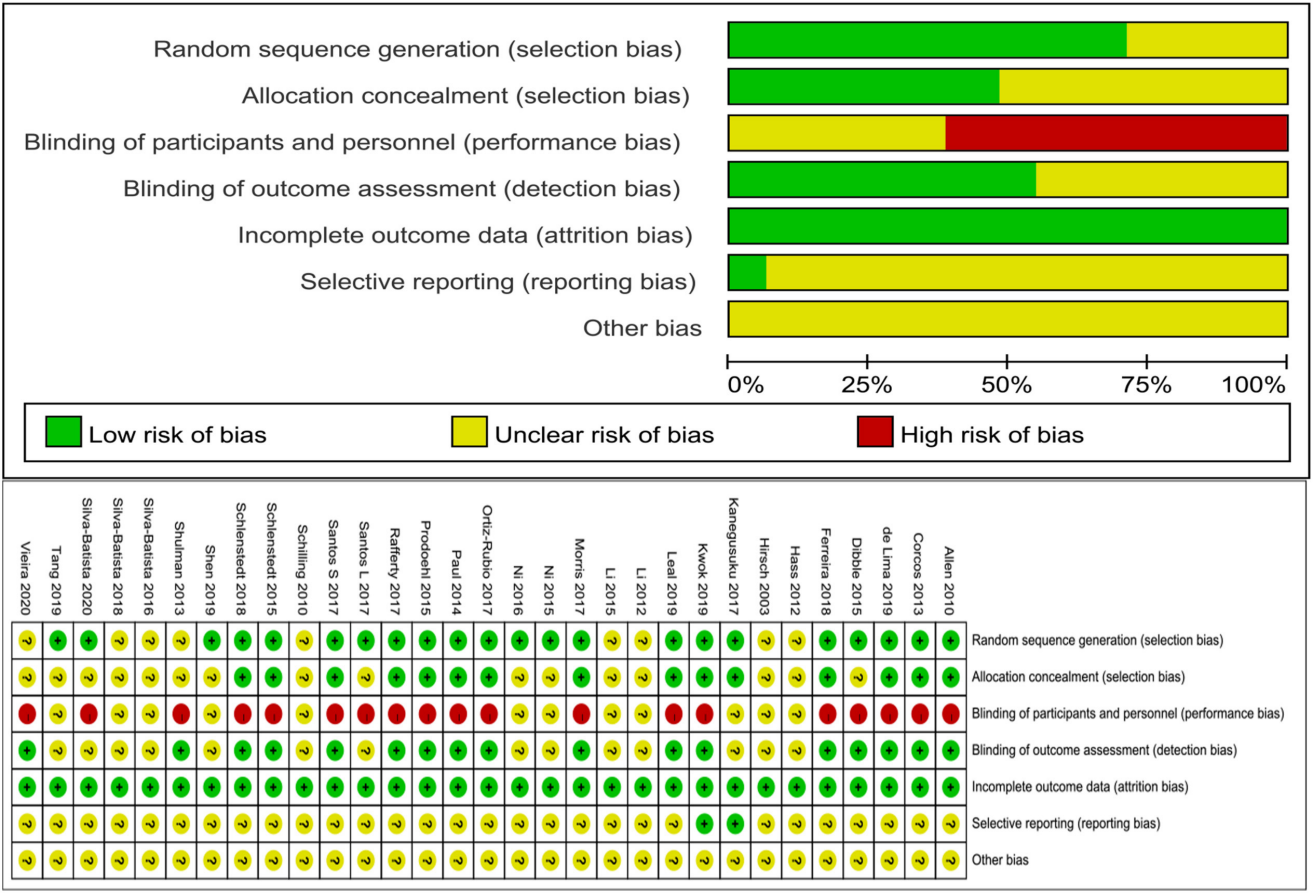
Quality of the included articles by the bias risk assessment based on the Cochrane collaboration Network.

Thirty one papers compromising 1,239 subjects were selected for eligibility in this systematic review and meta-analysis (7, 9, 10, 13, 16, 17, 23, 26–49). Table 1 contains the general characteristics of the included articles between the experimental groups and controls in individuals with PD. These articles were all RCTs and published from January 2000 to July 2020. The bias risk assessment results of the included articles are presented in Figure 2. Fifteen of the studies with target population were from the Americas (8 from North America and 7 from South America), 4 studies were from Europe, 3 studies were from Oceania, and 3 studies were from Asia. Twenty nine of these studies were published in English and 2 were published in Chinese. The sample size of each publication varied between a low of 15 and a maximum of 138. The average age of included subjects from each article varied from 58.8 to 79.5. The medical diagnosis, disease stage, and inclusion and exclusion criteria of patients were adequately reported among the included studies. Subjects involved in these studies were mostly mild to moderate PD according to the Hoehn and Yahr Staging Scale of 1–4. To further explore extreme heterogeneity, we performed a detailed descriptive analysis.

**Table 1:** General characteristics of these included articles concerning the effects of lower limb RT in individuals with PD.

**References**	**Year**	**Study design**	**Sex ratio (M/F)**	**Age (Mean ± SD)**	**N (E/C)**	**Hoehn-Yahr Stage + duration (*n*)**	**Frequency+intervention time**	**Follow-up period**	**Outcome measures and relevant parameter changes**	**Group Training**	
										**RT**	**Control**
Silva-Batista et al. (27), Brazil	2020	RCT	21/11	65.6 ± 9.7	17/15	3~4; 8.8 ± 4.9	80~90 mi, 3/wk; 12 wk	12 wk	1.FOGQ(↓); 2.PDQ-39(↓).	Lower and upper limb PRT. ^*^Half-squat; plantar flexion; knee lifting stand; lunge; reverse fly; dual-task squat.	Traditional motor
Vieira De Moraes Filho et al. (17), UK	2020	Single blinded RCT	30/10	64.6 ± 2.6	25/15	1~3; 6.3 ± 1.5	50~60mi, 2/wk; 9 wk	9 wk	1.TMWT(↑); 2.TUG(↓).	Lower limb and trunk RT. ^*^Knee extension; hamstrings curl; leg press; seated row.	No intervention
Kwok et al. (16), Hong Kong	2019	Single blinded RCT	65/73	63.6 ± 8.7	67/71	1; NR	60 mi, 2/wk; 8 wk	0, 8, 20 wk	1.TUG(↓); 2.PDQ8(↓).	Lower and upper limb SRTE. ^*^Resistance training and stretching.	Mindfulness yoga
de Lima et al. (10), Brazil	2019	Single blinded RCT	NR	66.7 ± 5.3	17/16	1~3; NR	30~40mi, 2/wk; 20 wk	20 wk	1.PDQ39(↓); 2.Gait velocity(↑); 3. TUG(↓).	Lower and upper limbs RT. ^*^ Bench press, unilateral rowing, standing calf raise, and abdominal reverse crunch.	No intervention
Shen et al. (28), China	2019	RCT	47/38	65 ± 4.8	40/45	1~4; 4.6 ± 1.9	NR; 18 mo	0,3,12,18 mo	BBS(↑).	Lower and upper limb PRT.	No intervention
Tang et al. (23), China	2019	RCT	43/19	68.7 ± 5	31/31	1~3; 4 ± 2.2	20 mi, 2/wk; 12 wk	12 wk	1.Muscle strength(↑); 2.BBS(↑).	Medium and low intensity lower limb PRT. ^*^Elastic band leg lift; lunge with elastic band; side pull of elastic band; seated leg lifts.	No intervention
Leal et al. (29), Brazil	2019	Single blinded RCT	27/27	65.1 ± 2.2	27/27	1~3; NR	20 mi, 2/wk; 6 mo	6 mo	1.TMT-B(↑); 2.6MWT(↑); 3.TUG(↓).	Low-volume lower and upper limb RT. ^*^Bench press; deadlift; unilateral rowing; standing calf raise; abdominal reverse crunch.	No intervention
Schlenstedt et al. (30), Germany	2018	Single blinded RCT	15/5	79.5 ± 6.4	12/8	2.5~3; 10.1 ± 6.8	60 mi, 2/wk; 7wk	8, 12 wk	1.FAB(↓); 2.FOGQ(↓).	Moderate frequency lower limb RT. ^*^Squats; knee extensions; toe/calf raises; hip abductions.	Balance training
Ferreira et al. (9), Brazil	2018	Single blinded RCT	NR	65.8 ± 8.1	18/17	1~3; 5.5 ± 3.5	30~40mi, 2/wk; 24 wk	24 wk	PDQ39(↓).	Lower and upper limb RT. ^*^Bench press; unilateral rowing; standing calf raise.	No intervention
Silva-Batista et al. (7), Brazil	2018	RCT	19/7	64.2 ± 8.5	13/13	2~3; 10.2 ± 5.1	60 mi, 2/wk; 12 wk	12 wk	BESTest(↑)	Lower and upper limb PRT. ^*^Half-squat; plantar flexion; leg-press.	No intervention
Morris et al. (31), Australia	2017	Single blinded RCT	80/53	71 ± 9	67/66	1~4; NR	60 mi, 1/wk; 6 wk	12 mo	PDQ-39(↑).	Lower limbs and trunk PRT. ^*^Step-ups; heel raises; sit-to-stand movements; standing hip abduction exercises.	No intervention
Santos S et al. (32), Brazil	2017	Single blinded RCT	28/12	67.8 ± 7.1	19/21	1.5~3; 5.5 ± 4.8	60 mi, 2/wk; 8 wk	8 wk	BESTest(↓).	Lower limbs and trunk RT. ^*^Muscular strengthening; stretching training.	Balance training
Santos L et al. (26), Spain	2017	RCT	15/13	73.6 ± 7.8	13/15	1~2; 10.6 ± 4	60~70mi, 2/wk; 8 wk	0, 8, 12 wk	1.TMWT(↑); 2.FOGQ(→); 3.PDQ39(↓); 4.Stride length(↓).	Lower and upper limb PRT. ^*^Knee extension; knee flexion.	Activity routine
Ortiz-Rubio et al. (33), Spain	2017	Single blinded RCT	33/13	74.8 ± 6.1	23/23	2~3; 4.1 ± 2	60 mi, 2/wk; 8 wk	8 wk	Mini-BES Test(↑).	Lower limb PRT. ^*^Strengthening all major muscle power seated with elastic bands.	Seated stretching
Rafferty et al. (34), USA	2017	Single blinded RCT	28/20	58.8 ± 5.1	24/24	2.5~3; 6.5 ± 4.7	60~90mi, 2/wk; 24 mo	6, 12, 18, 24 mo	Stride length(↓).	Trunk, lower and upper limb PRT. ^*^Double leg press; hip extension; rotary calf; seated quadriceps extension.	MFCE
Kanegusuku et al. (35), Brazil	2017	RCT	22/5	65.2 ± 8.1	15/12	2~3; 8.7 ± 4.6	30~60mi, 2/wk; 12 wk	12 wk	1RM(↑).	Lower and upper limb PRT. ^*^Horizontal leg press, squat, rotary calf, 17 lateral pull down	No intervention
Silva-Batista et al. (36), Brazil	2016	RCT	19/7	64.2 ± 8.5	13/13	2~3; 10.2 ± 5	60 mi, 2/wk; 12wk	12 wk	1.TUG(↑); 2.PDQ39(↓); 3.1RM(↑).	Lower and upper limb PRT. ^*^Leg-press; latissimus dorsi pull-down; ankle plantar flexion; half-squat.	No intervention
Ni et al. (37), USA	2016	RCT	13/11	73 ± 7.4	14/10	1~3; 6.3 ± 5.1	45~60mi, 2/wk; 12 wk	12 wk	1.Muscle strength(↑); 2.PDQ39(↓).	Lower and upper PWT. ^*^Leg press; leg curl; hip abduction; seated calf.	YOGA
Li et al. (13), USA	2015	RCT	77/53	69 ± 8.5	65/65	1~4; 7 ± 7.3	60 mi, 2/wk; 24 wk	24 wk	PDQ8(→).	Lower limb PRT. ^*^Forward and side steps; squats; forward and side lunges; heel and toe raises.	Stretching
Ni et al. (38), USA	2015	RCT	20/7	71.4 ± 6.4	14/13	1~3; 6.7 ± 5.3	45~60mi, 2/wk; 12 wk	12 wk	1.BBS(↑); 2.TUG(↑); 3.Mwalk(↓); 4.1RM(↑↓→).	Lower and upper limb PWT. ^*^Leg press; leg curl; hip abduction; seated calf.	YOGA
Dibble et al. (39), USA	2015	Single blinded RCT	25/16	68.4 ± 12.3	20/21	2~4; 6.8 ± 4.5	60 mi, 2/wk; 12 wk	12 wk	1.Leg strength(↓); 2.6MWT(↓).	Lower limb RT. ^*^Training of bilateral lower extremity extensor musculature.	Regular exercises
Prodoehl et al. (40), USA	2015	Single blinded RCT	28/20	58.8 ± 5.1	24/24	NR; 6.5 ± 4.4	60~90mi, 2/wk; 24 mo	6, 24 mo	1.TUG(↑); 2.BBS(↓); 3.6MWT(↓).	Upper and lower limb PRT. ^*^Double leg press; hip extension; rotary calf; seated quadriceps extension.	MFCE
Schlenstedt et al. (41), Germany	2015	Single blinded RCT	21/11	75.7 ± 6.3	17/15	2.5~3; 9.7 ± 6.9	60 mi, 2/wk; 7wk	8, 12 wk	1.FAB(↓); 2.TUG(↑); 3.Stride length(↓) and gait velocity(↓); 4.Leg strength(↓); 5.PDQ39(↓).	Lower limb RT. ^*^Squats; knee extensions; toe/calf raises; hip abductions.	Balance training
Paul et al. (42), Australia	2014	Single blinded RCT	25/15	66.3 ± 6.7	20/20	1~3; 7.8 ± 5.5	45 mi, 2/wk; 12 wk	12 wk	1.Muscle strength(↑); 2.TUG(↓); 3.FOGQ(↓); 4.Gait velocity(↑); 5.Balance(↑).	Lower limb RT. ^*^Leg extensors; knee flexors; hip flexors; hip abductors.	Sham exercise
Shulman et al. (43), USA	2013	Single blinded RCT	34/10	65.6 ± 11.3	22/22	1~3; 6.3 ± 3.7	30 mi, 3/wk; 3 mo	3 mo	1.6MWT(↓); 2.1RM(↑).	Lower limb RT. ^*^Hip abduction; stretches of calves, hamstrings, quadriceps, and ankles.	Treadmill exercise
Corcos et al. (44), USA	2013	Single blinded RCT	28/20	58.8 ± 5.1	24/24	2.5~3; 6.5 ± 4.4	60~90mi, 2/wk; 24 mo	6,12,18,24 mo	PDQ-39(↓).	Lower and upper limb PRT. ^*^Double leg press; hip extension; rotary calf (ankle plantar flexion; seated quadriceps extension.	MFCE
Li et al. (45), USA	2012	RCT	77/53	69 ± 8.5	65/65	1~4; 7 ± 7.3	60 mi, 2/wk; 24 wk	24 wk	1.Stride length(↑); 2.Gait velocity(↑); 3.TUG(↓).	Lower limb PRT. ^*^Forward and side steps; squats; forward and side lunges; heel and toe raises.	Stretching
Hass et al. (46), USA	2012	RCT	14/4	65.5 ± 7.5	9/9	1~3; 8.8 ± 7.4	60 mi, 2/wk; 10 wk	10 wk	1.Stride velocity(↑); 2.Stride length(↓); 3.1 RM(↑).	Lower limb PRT. ^*^Seated leg press; knee extension;knee flexion; seated calf raise; multi-directional seated ankle theraband.	No intervention
Allen et al. (47), Australia	2010	Single blinded RCT	26/22	67 ± 8.6	24/24	NR; 8 ± 5.6	40~60mi, 3/wk; 6 mo	6 mo	1.Muscle Strength(↑); 2.BBS(↓); 3.FOGQ(↓); 4.Gait velocity(↑); 5.PDQ39(↓).	Lower limb PRT. ^*^Stepping and standing; walking; sit-to-stand; heel raises; lateral and forward step-up, half-squats sliding; standing on one leg.	No intervention
Schilling et al. (48), USA	2010	RCT	11/7	59.3 ± 8	9/9	1~2.5; NR	40 mi, 2/wk; 8 wk	8 wk	[n] RM(↑); 2.TUG(↓); 3.6MWT(↑); 4.ABC(↑).	Moderate volume, high-load, lower limb RT. ^*^Leg press; leg curl; calf press.	No intervention
Hirsch et al. (49), USA	2003	RCT	NR	73.7 ± 3.3	6/9	1~3; 7.2 ± 7.9	15 mi, 3/wk; 10 wk	10,14 wk	1.EquiTest(↑); 2.Muscle Strength(↑).	High-intensity lower limb PRT. ^*^Ankle plantarflexors; knee extensors; flexors.	Balance training

*↑, increase; ↓, decrease; → , no changes; RT, Resistance training; PRT, Progressive resistance training; BT, Balance training; PWT, Power training; SRTE, Stretching and resistance training exercises; NR, Not reported; mi, minute; wk, week; mo, month; MFCE, Modified fitness counts exercise; TUG, Timed-up-and-go-test; FOG-Q, Freezing of Gait Questionnaire; QoL, Quality of life; PDQ-39, Parkinson's Disease Questionnaire; 1-RM, One repetition maximum; MWT, Meter walking tests; BBS, Berg Balance Scale; FAB, Fullerton Advanced Balance; BESTest, Balance Evaluation systems test; ABC, Activities-specific Balance Confidence*.

### Meta-Analysis Results

#### Leg Strength

**Figure 3:**
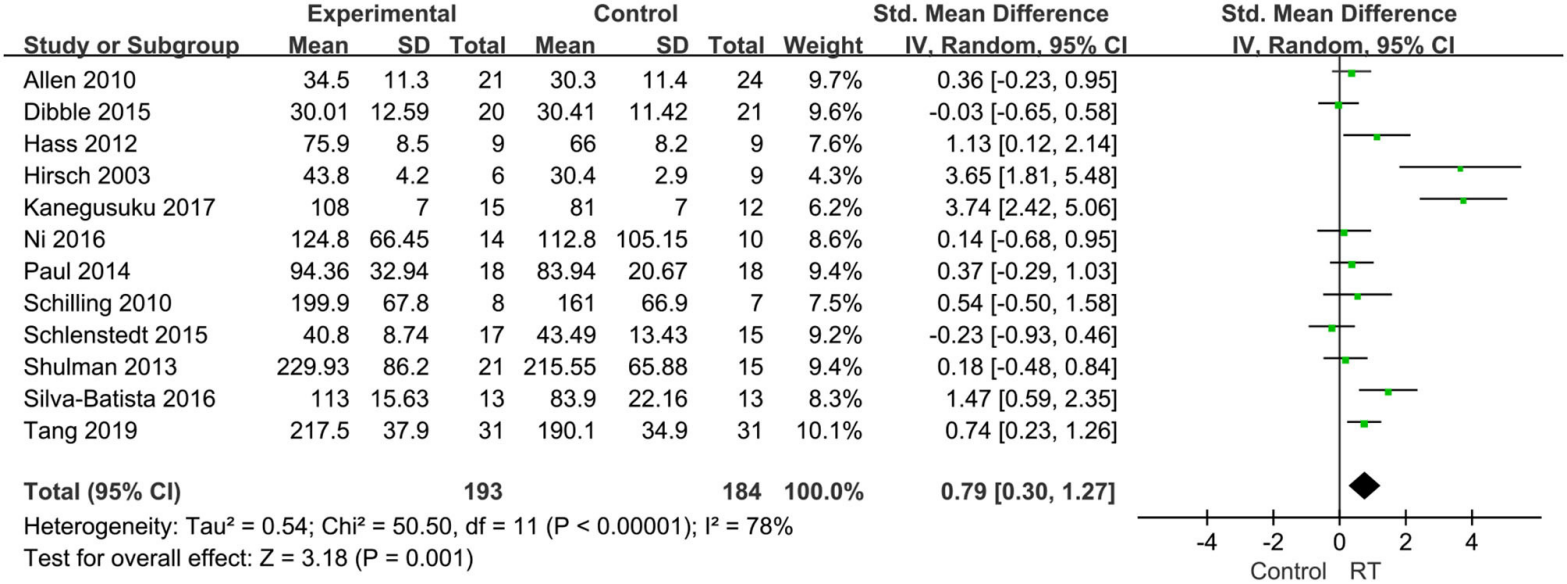
Forest plots of the effects of lower limb RT on leg strength in PD. CI, confidence interval.

Among the included publications, 13 discussed muscle strength of the lower limbs, and two articles (37, 38) were from the same study, so we adopted the data from the latest one. Therefore, 12 articles involving 377 subjects informed the analysis. Summarized data presented that the leg strength increased significantly in the experimental group compared to the control group (SMD = 0.79, 95% CI 0.3, 1.27, *P* = 0.001), indicating a positive effect of RT on strength in the lower limbs. In consideration of high heterogeneity between articles (*P* < 0.00001, *I*^2^ = 78%), a random-effects model was conducted to pool the data (Figure 3).

#### Balance Capability

**Figure 4:**
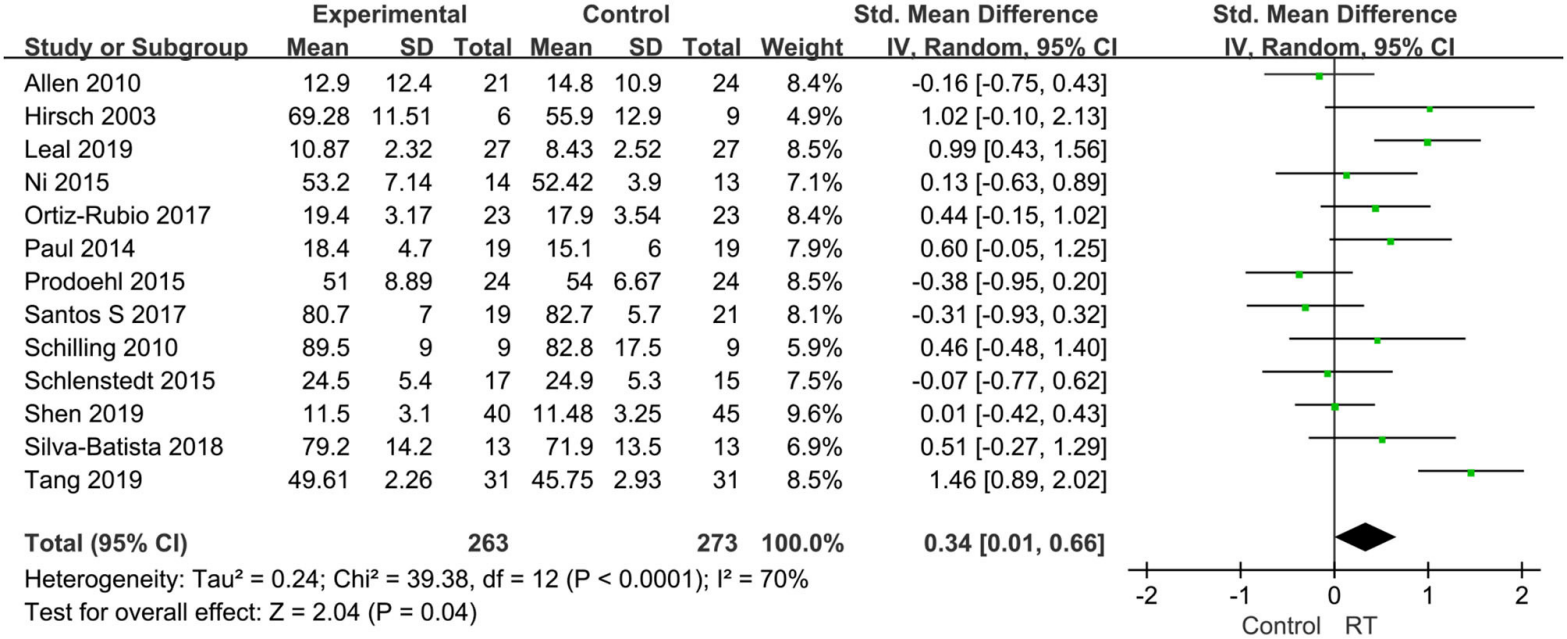
Forest plots of the effects of lower limb RT on balance performance in PD. CI, confidence interval.

Fourteen of all included literature reported the effectiveness of RT on balance performance in individuals with PD, while two papers (30, 41) reported on the same study, we chose the article with the larger sample size for this analysis. Therefore, there were 13 articles compromising 536 patients involved in the analysis. The pooled effects displayed that balance capability in experiment group improved after the RT compared to that in the control group (SMD = 0.34, 95% CI 0.01, 0.66, *P* = 0.04). There was moderate heterogeneity between studies (*P* < 0.0001, *I*^2^ = 70%) and a random-effects model was used to pool the data (Figure 4). These results suggested that RT could be beneficial for improving balance in PD.

#### Gait Performance

**Figure 5:**
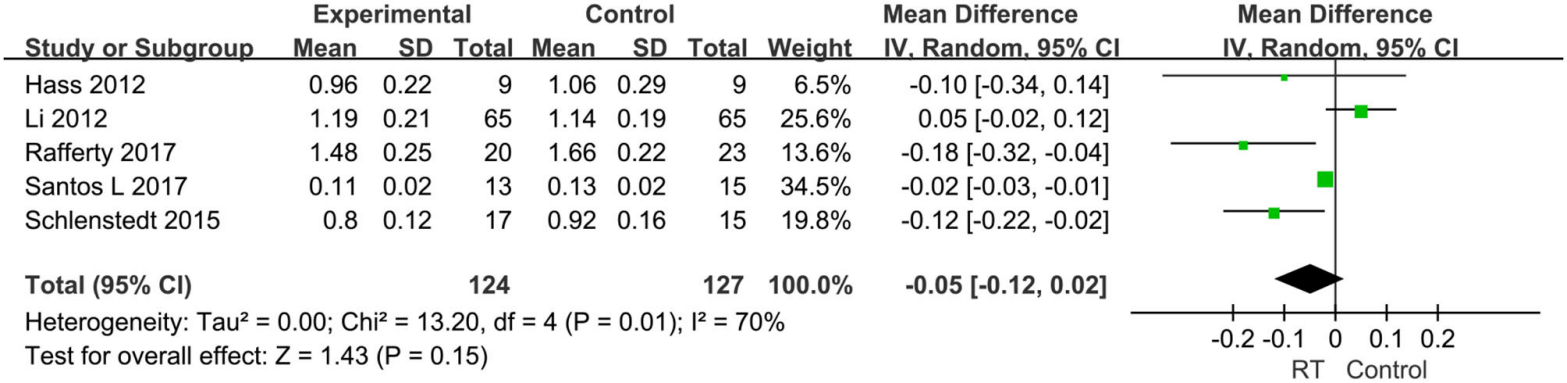
Forest plots of the effects of lower limb RT on gait performance—stride length in PD. CI, confidence interval.

**Figure 6:**
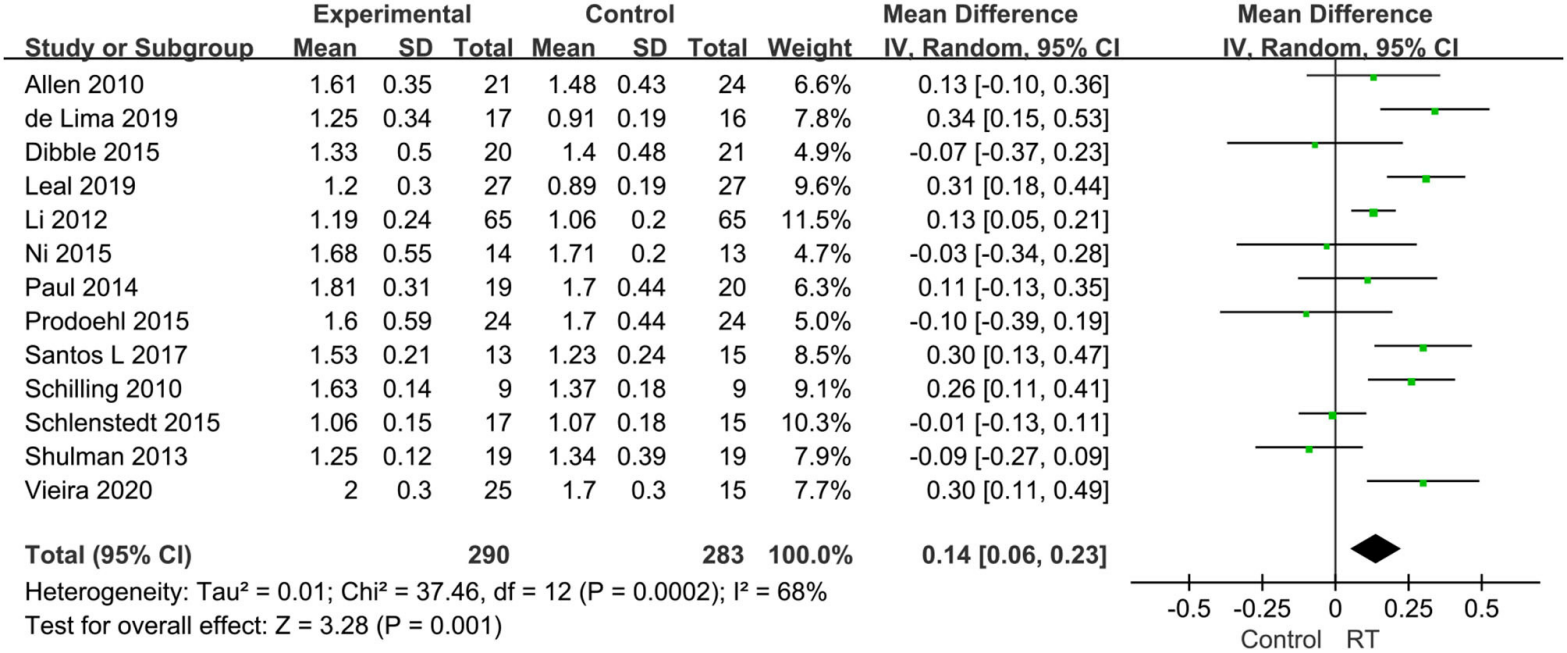
Forest plots of the effects of lower limb RT on gait performance—fast gait velocity in PD. CI, confidence interval.

**Figure 7:**
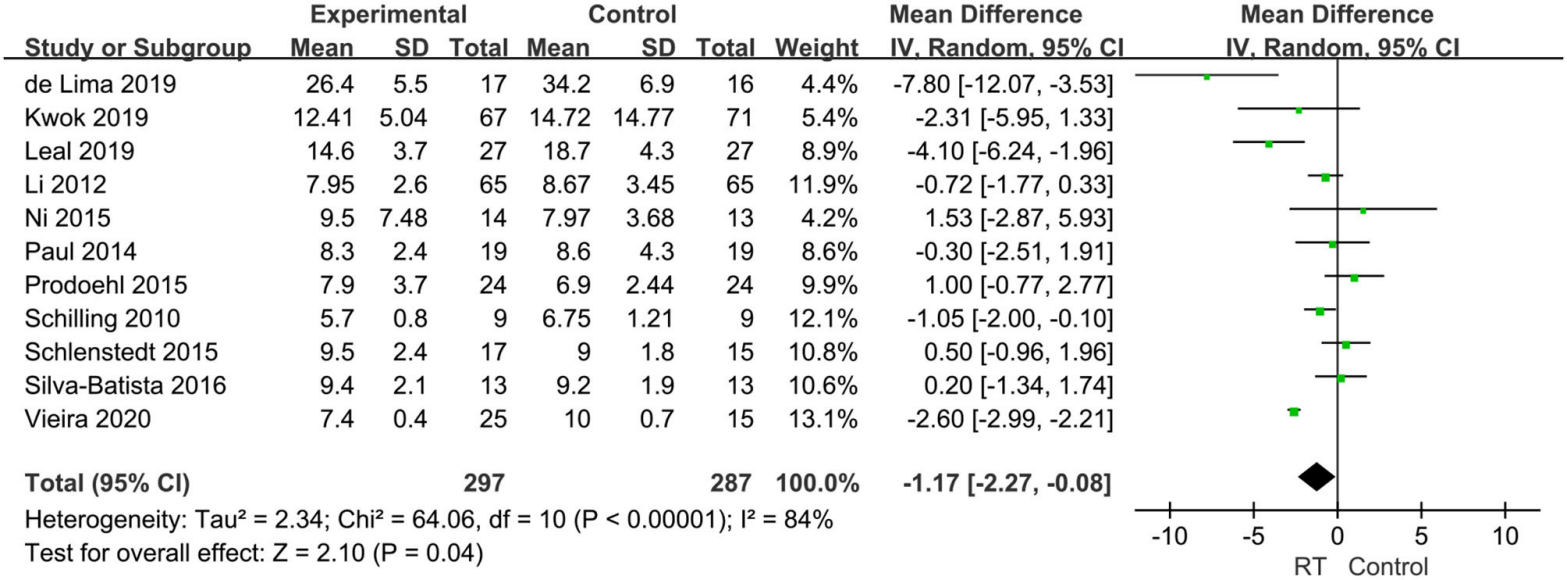
Forest plots of the effects of lower limb RT on gait performance—timed-up-and-go-test in PD. CI, confidence interval.

**Figure 8:**
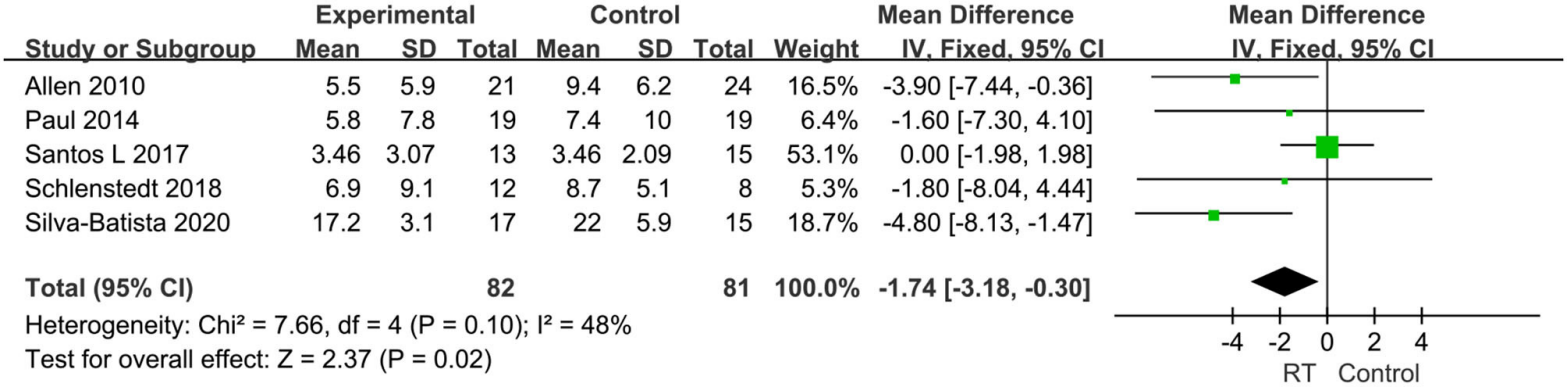
Forest plots of the effects of lower limb RT on gait performance—freezing of gait in PD. CI, confidence interval.

Eighteen articles compromising 818 subjects evaluated the effects of RT on gait performance in PD, and mainly focused on the following four aspects: stride length, fast gait velocity, TUG, and FOG-Q. ① Stride length was measured in five articles with 251 subjects. We set the parameter m as the unified unit of measurement. Pooled effects revealed that the stride length was not significantly different between the pooled experimental group and control group (MD = −0.05, 95% CI −0.12, 0.02, *P* = 0.15), with high heterogeneity between studies (*P* = 0.01, *I*^2^ = 70%) by a random-effects model to obtain the results (Figure 5). ② Fast gait velocity was measured in 13 articles with 573 subjects, and we set the parameter m/s as the unified unit of measurement. The pooled effects displayed that the fast gait velocity was significantly different between the pooled experimental group and control group (MD = 0.14, 95% CI 0.06, 0.23, *P* = 0.001), with moderate heterogeneity between studies (*P* = 0.0002, *I*^2^ = 68%) by a random-effects model to obtain results (Figure 6). ③ The TUG was measured in 11 articles compromising 584 patients, and we set the parameter s as the unified unit of measurement. The pooled effects demonstrated that the TUG time in experimental group was significantly quicker than in the control group (MD = −1.17, 95% CI −2.27, −0.08, *P* = 0.04) with high heterogeneity between studies (*P* < 0.00001, *I*^2^ = 84%) requiring a random-effects model to determine the results (Figure 7). The high heterogeneity could be related to different stages of disease and non-uniform training programs. ④ The FOG-Q was measured in 5 articles compromising 163 patients. The pooled effects showed that there was a significant difference in FOG-Q score between the experimental group and the control group (MD = −1.74, 95% CI −3.18, −0.3, *P* = 0.02) with low heterogeneity between studies (*P* = 0.1, *I*^2^ = 48%) allowing a fixed-effects model to determine the result (Figure 8). These indicators of gait performance indicated that RT could improve fast gait velocity, TUG and freezing of gait, but potentially not stride length.

#### Quality of Life (QoL)

**Figure 9:**
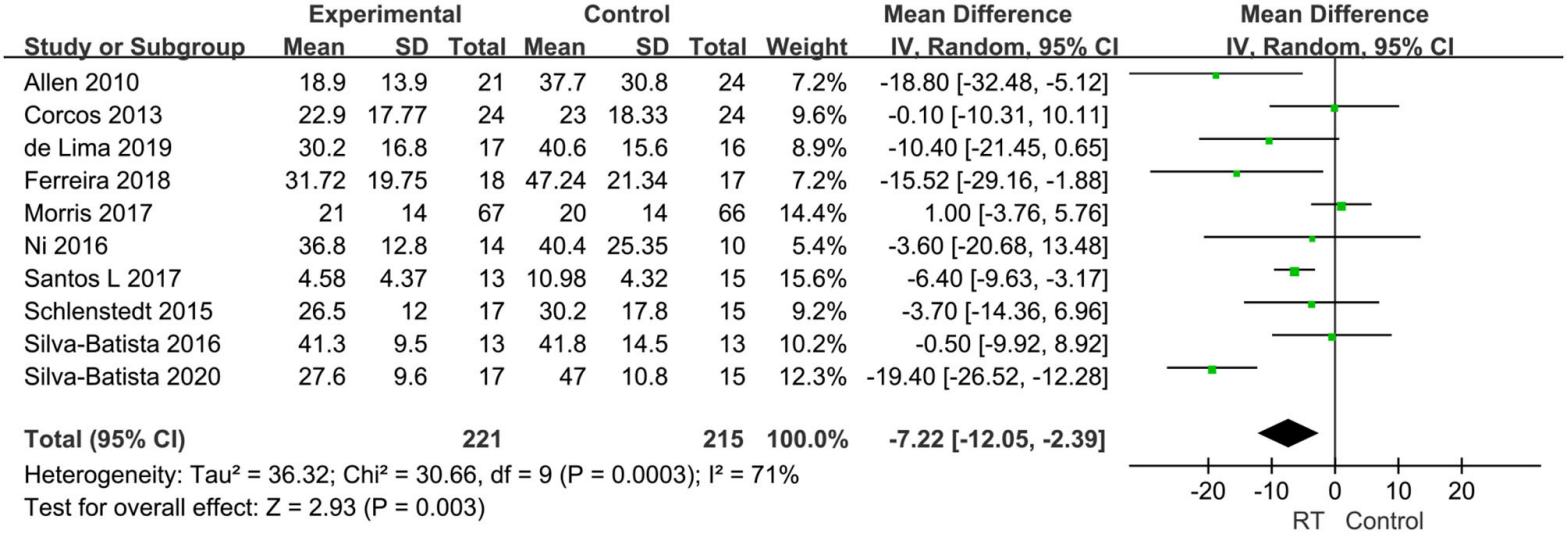
Forest plots of the effects of lower limb RT on quality of life in PD. CI, confidence interval.

There were 12 articles measuring health-related QoL in subjects with PD. The score item in one study was the Parkinson's Disease Questionnaire (PDQ-8), and in one another study was a disease-specific 8-item PDQ (Chinese version). All other studies used the PDQ-39. To ensure the consistency of results, we combined the data from the PDQ-39 only. Ten studies with 436 subjects were involved in this analysis. The pooled effects analysis revealed that patients in the experimental group had significantly lower PDQ-39 scores than in the control group (MD = −7.22, 95% CI −12.05, −2.39, *P* = 0.003) with moderate heterogeneity between studies (*P* = 0.0003, *I*^2^ = 71%) requiring a random-effects model to determine the result (Figure 9), suggesting that RT has a positive effect on the QoL in individuals with PD.

### Sensitivity Analysis and Publication Bias

**Figure 10:**
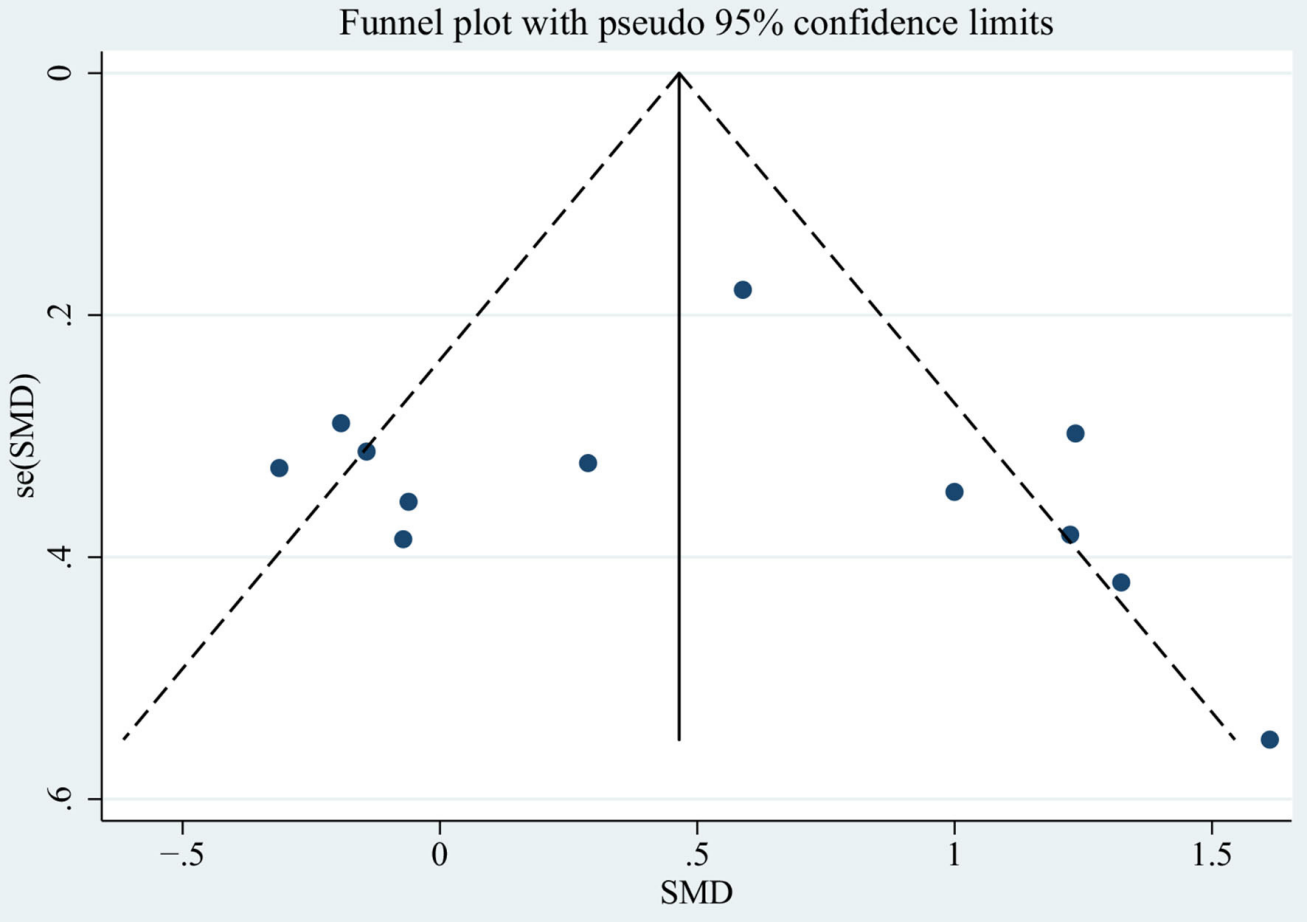
Funnel plot for the assessment of publication bias.

We used the fast gait velocity analysis, which included in the most literature, as an example to conduct sensitivity analysis and publication bias. Sensitivity analyses suggested that no single study could affect the total effect size by omitting the articles one-by-one. The shape of the funnel plot (Figure 10) was generally symmetric in the overall publication, the data points were equally distributed around the funnel and no obvious small sample effects. These showed that no indication of significant publication bias was, respectively, found which is consistent with the Begg's test (*P* = 0.3 > 0.05) and Egger's test (*P* = 0.621 > 0.05) in the overall population.

## Discussion

In this updated systematic review and meta-analysis of RCTs, 31 eligible articles from 25 independent trials compromising 1,239 subjects were summarized to verify the effects of lower limb RT in PD. In general, the synthesis of the existing research identified positive effects in PD symptoms with RT. Our major findings suggested significant improvement in balance capacity, gait performance, and QoL, which appeared contradictory to previous systematic reviews (24, 25). This mainly due to that previous studies with limited studies were involved, while studies included in our study with larger sample size and wider population.

The results of our study were reliable and robust from the perspective of systematic review methods, which included the latest RCTs and more comprehensive evidence of the topic, with more diverse geographical areas covering North and South America, Europe, Asia, and Oceania, with a relatively larger sample size (*n* = 31), and the Cochrane risk of bias tool was used for quality assessment among included RCTs. Eighteen of the 31 included articles were single-blinded RCTs, and blinded assessors or blinded physiotherapists were engaged in the intervention process to ensure concealment of allocation or outcome assessment, which considering the nature of motor intervention that it would be hard to blind the subjects. It's not clear whether there is a practical blind method the remaining studies of both subjects and treatment teams in the therapeutic process. Thus, blinding seems to be the primary limitation in design and method of these current studies.

From the pooled analysis, it was concluded that RT could significantly increase leg strength in individuals with mild-to-severe PD over periods of 8 weeks−24 months. The results agree with the latest meta-analyses by Tillman et al. (24) and Roeder et al. (50). The main mechanism responsible is likely that RT results in strong stimulation of skeletal muscle protein synthesis, promotes muscle cell growth, improves skeletal muscle quality, causes cyclic assimilation hormone to induce increases in absolute muscle strength and muscle cross-sectional area ratios, and optimizes skeletal muscle innervation and activation, thus enhancing lower limb muscle strength in patients with PD (23, 50, 51). Muscle strength of the lower limbs in patients with PD is markedly reduced, which is related to mobility, falls risk (39) and increased mortality (35). Although training programs differed widely among the included studies (frequency: 15–90 min/session, 1–3 sessions/week; intensity: low-to-high volume; and duration: 6 weeks−24 months), all protocols were effective in improving strength, power and physical performance in older patients. Previous studies have suggested patients with PD experienced highly varied strength gains though RT, which could be affected by disease severity, baseline muscle strength or intensity, intramuscular fat, and neural activation (52, 53). While a recent systematic review by Lopez et al. (54) hold that no association between exercise type, RT duration, weekly volume and intensity, and fitness outcomes (i.e., muscle strength) in men with prostate cancer. Consistent with our results, Douris et al. (55) indicated a significant increase in leg strength in a recreationally active person with PD and found decreases in restless leg syndrome after 18-weeks of RT exercises.

Postural instability is a common symptom in patients with PD and is considered to be the most significant factor related to falls and increased morbidity (56). Current literature on the effects of RT on postural balance disorders in PD remains controversial; a meta-analysis by Chung et al. (25) demonstrated that RT can result in significant balance gains in mild-to-moderate PD, while Tillman et al. (24) suggested no discernable effect of RT on balance measures. Our study showed that the balance capacity of patients in the experimental group was better than that before exercise following training. Several plausible reasons could account for this: RT could increase the muscle strength and explosive force of the quadriceps femoris in patients with PD, improve the stability of knee joint, improve the strength and stability of the trunk muscles, regulate the sensitivity of the neuromuscular control system and sensation, improve neuromuscular control efficiency and posture control ability, and improve patients' balance ability. Hewitt et al. (8) supposed that after RT, PD patients in the experimental group improved in physical performance and functional mobility, with reduced falls and fall rates. In line with our results, significant improvements in balance-related activities of home-based prescribed exercise were also verified in another recent systematic review by Flynn et al. (57). We acknowledged that balance control is complex and involves many different underlying systems, many clinical tests were designed to use one single “balance system” and used one or two balance measuring tools, which themselves varied greatly. And since the subjects of included studies did not classify in different phenotypes, i.e., tremor dominate (TD), postural instability/gait disorders (PIGD), so it is impossible to know which phenotype in PD is more effective in balance capacity with RT. Additionally, one recent publication (58) have highlighted the variability in postural instability by genetic subtypes; given phenotypic severity with wide range and genetic data is increasingly more important than phenotypes, the included articles in our study did not assess greater granularity in genetic. The lack of uniformed balance measures, motor phenotypes and genetic analysis reported in RT intervention studies limits our analysis, we suggest the results may not be suitable for every PD patients with caution and there remains a need for further research to confirm this conclusion.

Gait performance is another crucial function of the lower limbs which usually affected by functional impairments in PD. Gait impairments are composed of motor defects, bradykinesia, stiffness, the postural instability characteristic of PD, and reduced the QoL. A prior review by Chung et al. (25) found no statistically significant effects of RT in patients with PD for gait performance, but our study suggested that RT could improve gait performance to a certain extent in PD. To understand the effects of RT on gait performance, we considered the following four subcategories: stride length, fast gait velocity, TUG, and FOG-Q. This study found that RT resulted in greatly significant differences in fast gait velocity, TUG, and FOG-Q between experimental groups and control groups, but no significant differences in stride length. All the four indicators were specific, utilized a unified measurement tool, or could be unified through calculation, so that the results of analysis were robust. Therefore, it is reasonable to believe that the RT has positive effects on gait performance to a certain extent in patients with PD, especially in fast gait velocity, freezing of gait, and functional walking capacity. The results in the studies by Dibble et al. (59) and Silva-Batista et al. (18) were similar to the effects of our study in that they demonstrated that outcome variables and gait performance measures were greatly improved after RT exercise compared to the control groups.

With respect to the effect of RT on the QoL, the former review by Chung et al. (25) found no significant improvement in PD, whereas our study found that the QoL score among patients in the experimental group was significantly decreased compared to the control group and therefore shows positive change. To assess QoL among patients with PD, we adopted a unified indicator through the PDQ-39, which is a disease-specific quality-of-life instrument for use in PD. In a recent study by Hewitt et al. (8), a significant improvement in QoL in patients with PD resulted after a 25-week period of RT plus balance exercise. Additionally, anxiety and depressive symptoms were positively correlated with the PDQ-39 scores of these patients, and they may negatively affect the QoL; as Ferreira et al. (9) and de Lima et al. (10) were showed reducing anxiety/depressive symptoms in people with PD was helpful in improving QoL, which may be a possible explanation for how RT positively affected the PD patients' QoL.

Nevertheless, there still exist several limitations that require consideration in interpreting the results of our meta-analysis. Firstly, most of the literature in this study were focused on mild to moderate PD, therefore the findings may not be generalizable to severe PD. Secondly, marked differences were present in the exercise protocols, including intensity, frequency and duration. Additionally, some control groups of included studies had exercise interventions and some did not. Those may have led to high heterogeneity of the results of our study. Finally, because of the nature of exercise interventions, it is impossible to blind subjects with respect to exercising and to the specific exercises they are performing; this may easily lead to selective bias.

## Conclusion

In consideration of the present evidence, lower limb RT is a useful tool and has positive effects during rehabilitation in individuals with PD, especially in leg strength and QoL, and could improve gait performance to a certain extent in PD. In addition, our study found a positive effect of lower limb RT on balance capacity; given lack of uniformed balance measures, motor phenotypes and genetic analysis reported in RT intervention studies though, further studies are needed to confirm the conclusion. Therefore, it would be necessary for patients to engage in RT regularly and lengthen follow-up times, so as to improve the QoL in patients with PD. While considering the heterogeneity of the present RT protocols, the conclusion about the effects of RT in PD need more high quality and large samples of RCT to confirm this.

## Data Availability Statement

The raw data supporting the conclusions of this article will be made available by the authors, without undue reservation, to any qualified researcher.

## Author Contributions

The topic was selected by XL and JH. Literature search and selection were conducted by XL and JH. The data were extracted and analyzed by JH and HQ. The rough manuscript was drafted by XL and JH. JY modified the paper. All authors checked the final version of the article.

## Conflict of Interest

The authors declare that the research was conducted in the absence of any commercial or financial relationships that could be construed as a potential conflict of interest.
